# Truro Nursing Association

**Published:** 1921-03-19

**Authors:** 


### Truro Nursing Association.

Truro has done very well in the past year. Under the provident system they can show over 600 subscribers, an increase of over 100, and they end the year with a small credit balance. The Mayor (Mr. N. B. Bullen), who presided at the annual meeting, was elected President, and it was decided to ask the labour organisations to appoint three ladies in place of those previously appointed, because the latter had not attended any meetings held during the year.

